# Digit ratio (2D:4D) and transgender identity: new original data and a meta-analysis

**DOI:** 10.1038/s41598-020-72486-6

**Published:** 2020-11-09

**Authors:** Eva-Maria Siegmann, Tobias Müller, Isabelle Dziadeck, Christiane Mühle, Bernd Lenz, Johannes Kornhuber

**Affiliations:** 1 Department of Psychiatry and Psychotherapy, Friedrich-Alexander University Erlangen-Nürnberg (FAU), Schwabachanlage 6, 91054 Erlangen, Germany; 2 Psychiatric Practice, Treibberg 5, 90403 Nuremberg, Germany; 3 Department of Addictive Behavior and Addiction Medicine, Central Institute of Mental Health (CIMH), Medical Faculty Mannheim, Heidelberg University, Heidelberg, Germany

**Keywords:** Psychology, Biomarkers, Endocrinology, Medical research

## Abstract

Previously reported associations between second-to-fourth digit length ratio (2D:4D), a proxy for prenatal androgen load, and transgender identity have been inconsistent. The objectives of the present study were to provide additional original data and an updated meta-analysis concerning this association. In a study of 464 participants, we compared the 2D:4D of transgender individuals with age- and sex-matched controls. Patients were recruited at a specialized psychiatrist’s medical office, whereas controls were hired via flyers, advertisements, and as convenience sample. A random-effects meta-analysis of the literature (17 samples, n = 3674) also quantifies the overall magnitude of the difference in 2D:4D between transgender individuals and controls. In our study providing new original data, we found a significantly higher (i.e. feminized) left-hand 2D:4D in the male-to-female transgender (MtF) identity [mean age: 32.3 (18; 61)] than in the male control group [mean age: 34.5 (18; 65)] with a Cohen’s d = 0.271. Concordantly, the meta-analytic results suggest a significant difference in 2D:4D among MtF individuals compared to male controls [g = 0.153; 95% CI (0.063; 0.243)], which was even more pronounced when individuals had been diagnosed by a clinician instead of self-identified as transgender [g = 0.193; 95% CI (0.086; 0.300)]. In both studies, no significant results were revealed for female-to-male transgender individuals [mean age: 26.1 (18; 53)] versus female controls [mean age: 27.2 (18; 55)]. This original investigation and the updated meta-analysis clarify the association between transgender identity and 2D:4D indicating the influence of prenatal androgen on the development of gender identity in subjects born as males.

## Introduction

Transgender identity or gender dysphoria are defined as experiencing an inconsistency between physical phenotype and one’s perceived gender^1^. Evidence suggests that the prevalence has increased over the last decades to up to 5–14 male-to-female transgender (MtF) individuals per 1000 adult males and 2–3 female-to-male transgender (FtM) individuals per 1000 adult females^2^. This growth in prevalence is possibly due to greater social acceptance, de-pathologization, and greater awareness of therapeutic options^2^. Despite the growing amount of research in this area, causal mechanisms are still unclear. Animal studies show an association between perinatal testosterone and the size of the bed nucleus of the stria terminalis (BNST)^3^, a region linked to gender identity in humans^4^. The theory of early organizational testosterone effects on gender identity is further supported by review evidence that transgender individuals’ brains show changes away from their natal sex and toward their perceived gender^5^. Additionally, studies examining subjects with congenital adrenal hyperplasia (CAH) or subjects with partial or complete androgen insensitivity syndrome (AIS/CAIS) suggest that (1) levels of gender dysphoria are higher when brain androgenization *mismatches* gender of upbringing and (2) that levels of gender dysphoria are lower when brain androgenization *matches* gender of upbringing^6^. For a more detailed description of gender dysphoria in patients with disorders of sex development and possible confounding variables, see Sadr et al.^6^. In humans, however, it is not possible to directly investigate the effects of prenatal androgens owing to ethical reasons and the long time period between the intrauterine window and the establishment of gender identity. Thus, this research area relies on proxies such as the second-to-fourth digit length ratio (2D:4D), a biomarker determined by the balance of prenatal testosterone to estrogen^7^, and therefore indicating the prenatal androgen load^8^. A lower 2D:4D reflects higher prenatal androgen exposure^9^ and hence, the 2D:4D is lower in males than in females^10,11^. It is frequently used to examine the link between prenatal androgen exposure and postnatal phenotypes including diseases, mental disorders, and behaviors reflecting gender bias or being influenced by first trimester organizational changes induced by testosterone/estrogen ratios^12^, such as alcohol addiction^13^, autism spectrum disorders^14,15^, sexual orientation^16^, the risk for different forms of cancer^17^, and physical prowess^18^. Concerning gender dysphoria and 2D:4D, contradictory evidence exists, which has been summarized in a meta-analytical commentary conducted in 2017^19^ where the authors report a small effect size [g = 0.190; 95% CI (0.076; 0.304)] indicating feminized 2D:4D of the right hand (but not the left hand) in MtF individuals and no associations at all in FtM individuals. This short systematic review included only few studies with rather small sample sizes or data retrieved from abstracts (which can be influenced by reporting bias), and it did not provide extensive meta-regression and subgroup analyses or distinguish between case–control and cohort studies. In 2020, a more recent meta-analysis^6^ reported significantly feminized 2D:4D in MtF individuals (d = 0.24). This analysis applied rather strict inclusion criteria (e.g. excluding child samples) and did also not distinguish between patient and cohort samples. The aim of the present research was to re-address this question by adding novel original data from a larger sample size and to subject the entire body of literature to a meta-analysis while taking potentially confounding factors into account. In previous meta-analytic research on 2D:4D and addiction, we found that the effects are more pronounced when cases have been defined via clinical diagnosis instead of via self-disclosure^13^. Therefore, we especially focused on the difference between transgender individuals diagnosed by a specialized clinician according to classification systems versus self-identification as transgender or gender nonconforming. Gender nonconformity extends the term of transgender individuals insofar that it includes other gender-variant identities or non-binary people, as well. For both the original research and the meta-analysis, we tested the hypothesis that 2D:4D is larger, i.e. feminized, in MtF individuals than in male controls. When comparing FtM individuals with female controls, we decided to follow an exploratory approach since previous (meta-analytical) evidence^6,19^ is not conclusive. Following the results of our own previous meta-analytic research on 2D:4D^13^, we defined a second hypothesis regarding the meta-analysis, namely, that the difference between transgender individuals and controls is more pronounced when the transgender person has been diagnosed by a specialized clinician.

### Aims of the study

We wanted to investigate the difference in the second-to-fourth digit length ratio between transgender individuals and control subjects. Therefore, we conducted an original case–control study with large sample size and subjected the entire body of literature to a meta-analysis.

## Study 1: new original data

This study investigated a possible association of 2D:4D and transsexuality in a large sample. It was part of the Finger Length in Psychiatry (FLIP) project^13,20–27^, a line of independent studies aiming to elucidate the role of prenatal sex hormone organization in the etiopathogenesis of psychiatric disorders in childhood, adolescence, and adulthood, such as addictive disorders^22,26,28,29,30^, suicide completion^31^, or behavioral symptoms in children^32^. Another goal was to establish prevention targets and strategies based on this model^33^.

### Materials and methods

#### Sample description

From 2016 to 2019, we recruited MtF and FtM patients and male and female control groups. In total, datasets of 110 MtF and 151 FtM patients diagnosed with *transsexualism* according to ICD-10^34^ and *gender dysphoria* according to DSM-5^35^ and 101 male and 102 female age-matched control subjects were analyzed. The patients were enrolled in the study by a psychiatrist specialized in transsexualism (T.M.) at a medical office in Nuremberg, Germany. The control subjects were hired via flyers and advertisements (Facebook advertisements, posts on black boards) or as convenience sample (neighborhood in Erlangen and Bergisch-Gladbach, Germany). They were recruited by a medical student (I.D.) in the Department of Psychiatry and Psychotherapy of the University Hospital Erlangen, Germany.

Inclusion criteria for both groups consisted of providing written informed consent, age ≥ 18 years, and body mass index (BMI) of 18.5–35.0 kg/m^2^. Exclusion criteria consisted of severe physical illness, the diagnosis of an androgen insensitivity syndrome or an adrenogenital syndrome, acute suicidality, pregnancy, breastfeeding, and the endocrine disorders Morbus Addison, Morbus Cushing, diabetes type 1 and type 2, and endocrine cancers. We did also not enroll control subjects taking regular medications (except for contraceptives), with any history of psychiatric in-patient treatment, or an ambulatory consultation due to a mental illness during the preceding 10 years.

#### Gender identity/Gender Dysphoria Questionnaire and Utrecht Gender Dysphoria Scale

Independent from each other, three study team members (T.M., C.M., B.L.) translated the 54 items of the Gender Identity/Gender Dysphoria Questionnaire for Adolescents and Adults (GIDYQ-AA; 27 for the male and 27 for the female questionnaire)^36^ and the 24 items of the Utrecht Gender Dysphoria Scale (UGDS)^37^ into German. Afterwards, discrepancies were discussed, and a single translation was selected in agreement. Whereas the original GIDYQ-AA uses a mean with low scores implying gender dysphoria, the original UGDS uses a sum score with high scores implying gender dysphoria. In order to increase comparability, we reversed the GIDYQ scores and converted item scores in the UGDS to mean values. Thus, higher scores of the gender dysphoria scales used in this study represent higher gender dysphoria. The minimum and maximum scores possible were 1 and 5 (similar to Deogracias et al.^36^). High internal consistencies (Cronbach’s alpha) were found for the German versions: (1) 0.984 for the male GIDYQ (*n* = 197); (2) 0.985 for the female GIDYQ (*n* = 238); (3) 0.989 for the male UGDS (*n* = 197); and (4) 0.981 for the female UGDS (*n* = 238). Separate Cronbach’s alpha values for transgender individuals and controls are provided in Supplementary Table S1 (online supplement). The Pearson correlations between GIDYQ and UGDS are r = 0.599 (*p* < 0.001) for the MtF sample (n = 96), r = 0.087 (*p* = 0.389) for the male sample (n = 101), r = 0.240 (*p* = 0.005) for the FtM sample (n = 136), and r = 0.474 (*p* < 0.001) for the female sample (n = 102).

#### Other questionnaires

We assessed the participants’ sexual orientation on a scale from 1 (= attracted only to women) to 7 (= attracted only to men). Following a classification proposed by Lawrence et al.^38^ we examined early vs. late onset gender dysphoria in transgender individuals by asking about the onset time point (before vs. after puberty) and about the subjects’ discomfort with the assigned gender at birth during their childhood (present vs. absent).

#### Second-to-fourth finger length ratio

Right and left hands of the participants were scanned on common document scanners. The lengths of the index and ring fingers (distance from the middle of the basal crease to the tip of the fingers) were quantified using the GNU Image Manipulation Program (GIMP 2.8; https://www.gimp.org). Each finger was measured three times by each of three independent raters (9 times in total). The raters evaluated the scans of patients and controls separately. The files were numbered consecutively (controls) or with a patient’s code. Thus, the raters were blind concerning the sex of the control or the patients’ status as MtF or FtM individual. 2D:4D values were calculated for the mean of right-hand and left-hand 2D:4D (M2D:4D), right-hand 2D:4D (R2D:4D), left-hand 2D:4D (L2D:4D), and differences between R2D:4D and L2D:4D (2D:4Dr-l) since low 2D:4Dr-l values have been associated with high prenatal testosterone load, as well^8^. We found high interrater reliabilities (two-way random interrater correlation coefficient; absolute agreement): (1) M2D:4D: *n* = 462, 0.990; (2) R2D:4D: *n* = 464, 0.986; (3) L2D:4D: *n* = 462, 0.983; and (4) 2D:4Dr-l: *n* = 462, 0.946.

#### Ethical standards

The study was approved by the Ethics Committee of the Medical Faculty of the Friedrich-Alexander University Erlangen-Nürnberg (FAU; ID 194_16 B). All participants provided written informed consent.

#### Statistical analysis

For each natal sex individually, we compared M2D:4D, R2D:4D, L2D:4D, and 2D:4Dr-l between the patient and the age-matched control groups using the Student’s *t*-test. Levene's test for homogeneity of variance was also used and if necessary, the degrees of freedom were adjusted. The χ^2^ test was used to evaluate differences in the frequency of nominal variables. Pearson’s correlations were used to investigate continuous relationships between the 2D:4D measures and GIDYQ and UGDS scores in each group. We present the data as means and standard deviations or relative frequencies. Statistical significance was determined at *p* < 0.05 (2-sided). The data were analyzed using IBM SPSS statistics Version 24 for Windows (SPSS Inc., Chicago, IL, USA).

### Results

#### Sociodemographic characteristics

The MtF group did not significantly differ from the male group with regard to age, months of employment during the previous year, BMI, or marital status (Table 1). We found significantly lower body height, body weight, and likeliness to live in a partnership in MtF than in male participants. The FtM group did not differ from the female group in terms of age, months of employment during the previous year, body height, likeliness to live in a partnership, or marital status. The FtM group showed a significantly higher BMI and a higher weight. MtF and FtM groups scored significantly higher on the GIDYQ and UGDS (suggesting higher gender dysphoria) than the male and female control groups.

**Table 1:** Group comparisons (Student’s *t* tests and χ^2^-tests): male-to-female versus male and female-to-male versus female groups.

	Male-to-female *N* = 110			Male *N* = 101			t, χ^2^	*P*	d	Female-to-male *N* = 151			Female *N* = 102			t, χ^2^	*P*	d
	*N*	Mean	SD	*N*	Mean	SD				*N*	Mean	SD	*N*	Mean	SD			
Age (years)	110	32.3	12.0	101	34.5	13.8	− 1.2	0.235		151	26.1	7.9	102	27.2	8.2	− 1.1	0.276	
Months of employment during the previous year	96	7.6	5.0	101	8.1	5.0	− 0.7	0.499		138	7.5	5.1	99	7.4	5.0	0.3	0.785	
Body mass index (kg/m^2^)	110	23.9	4.6	101	24.7	3.7	− 1.4	0.163		151	26.3	7.2	102	22.7	2.9	5.5	** < 0.0001**	
Height (cm)	110	178	8	101	183	6	− 5.1	** < 0.0001**		151	167	7	102	167	7	0.1	0.955	
Weight (kg)	110	75.7	15.5	101	82.7	11.7	− 3.7	** 0.0002**		151	73.7	21.0	102	63.4	8.9	5.3	** < 0.0001**	
Living in a partnership (%)	103	42.7		101	64.4		9.6	**0.002**		140	45.7		101	58.4		3.8	0.052	
Married (%)	103	25.2		100	26.0		< 0.1	0.902		140	7.1		102	11.8		1.5	0.217	
GIDYQ mean score^a^	96	3.9	0.3	101	1.1	0.1	77.8	** < 0.0001**		136	4.1	0.2	102	1.1	0.2	119.6	** < 0.0001**	
UGDS mean score	96	4.4	0.5	101	1.1	0.1	61.6	** < 0.0001**		136	4.7	0.3	102	1.5	0.4	72.7	** < 0.0001**	
M2D:4D	109	0.966	0.027	101	0.960	0.027	− 1.6	0.114	0.222	150	0.972	0.027	102	0.974	0.029	0.7	0.457	− 0.071
R2D:4D	110	0.963	0.030	101	0.959	0.028	− 1.0	0.314	0.138	151	0.972	0.028	102	0.975	0.030	0.9	0.392	− 0.103
L2D:4D	109	0.969	0.029	101	0.961	0.030	− 2.0	**0.04973**	0.271	150	0.971	0.029	102	0.974	0.032	0.6	0.578	− 0.098
2D:4Dr-l	109	− 0.007	0.021	101	− 0.003	0.021	1.4	0.155	− 0.190	150	0.001	0.021	102	0.002	0.024	0.3	0.746	− 0.044

*GIDYQ* Gender Identity/Gender Dysphoria Questionnaire (absolute range 1–5, higher scores indicate stronger dysphoria), *UGDS* Utrecht Gender Dysphoria Scale (absolute range 1–5, higher scores indicate stronger dysphoria), *2D:4D* second-to-fourth finger length ratio, *M2D:4D* mean of R2D:4D and L2D:4D, *R2D:4D *right-hand 2D:4D, *R2D* length of the right-hand index finger, *R4D* length of the right-hand ring finger, *L2D:4D* left-hand 2D:4D, *L2D* length of the left-hand index finger, *L4D* length of the left-hand ring finger, *2D:4Dr-l* difference between R2D:4D and L2D:4D, *d* Cohen’s d.

*p* < 0.05 in bold, ^a^reversed.

#### Second-to-fourth finger length ratio

We found higher M2D:4D, R2D:4D, and L2D:4D in MtF than in male participants and lower M2D:4D, R2D:4D, and L2D:4D in FtM than in female participants; however, only the higher L2D:4D in MtF than in male participants reached significance with an effect size of Cohen’s d = 0.271 (Table 1), a result that partly supported our first hypothesis. The GIDYQ scores correlated significantly negatively with M2D:4D and R2D:4D in the MtF group, positively with L2D:4D, and negatively with 2D:4Dr-l in the male control group. Moreover, the UGDS score correlated negatively with L2D:4D in the male control group (Supplementary Table S2). Our analyses revealed no significant association between the transgender individuals’ sexual orientation and M2D:4D, R2D:4D, L2D:4D, and 2D:4Dr-l (Supplementary Table S3). There is no significant difference in M2D:4D, R2D:4D, L2D:4D, and 2D:4Dr-l between early and late onset gender dysphoria in our sample (data not shown). This result needs to be interpreted cautiously since sample sizes in the subgroups were small. We report frequencies of early and late onset gender dysphoria in Supplementary Table S4 (online supplement).

### Discussion

We found significantly feminized 2D:4D in MtF individuals compared to male controls in the left hand, but not in the right hand and no significant results in FtM individuals versus female controls. However, the nonsignificant differences were in accordance with the prenatal androgen hypothesis. Comprising 464 participants, to our knowledge, this investigation presents the largest sample size among case–control studies of 2D:4D and transgender identity. Nevertheless, a power analysis by Voracek et al.^19^ underlying their meta-analytical effect size suggests that a sample size of at least 872 participants would be necessary to be able to detect small effects. Due to small prevalence rates of transgender identity, it is difficult to reach such numbers in clinical settings.

The correlational analyses concerning 2D:4D and the UGDS scores did not reveal any consistent pattern and remain contradictory. When examining the scatterplot of 2D:4D and the GIDYQ score, an inverted U-shaped relationship emerged. Comparable U-shaped associations have been reported in previous studies, such as with respect to 2D:4D and academic performance, altruism, or targeting reaction time (for an overview, see Tektas et al.^20^). Moreover, the low standard deviations (SDs) in both the GIDYQ and the UGDS could attenuate any association with 2D:4D, thus entailing non-significant results. The low SDs further bring small Cronbach’s alpha values (as can be seen in Supplementary Table S1, online supplement), indicating that the two scales do not differentiate well among members of the same group. Future research will be necessary to clarify the role of 2D:4D in the severity of gender dysphoria.

We used scanners to evaluate the subjects’ finger lengths. This method is said to produce lower 2D:4D values^39,40^, but to better reflect the sexual dimorphism^41^ when compared to direct measurement methods such as caliper. The high interrater correlation coefficients found in our study reflect the reliable accuracy of this method. Manning^12^ proposed that results are more precise when measuring 2D:4D directly than via scans, but a recent meta-analysis did not confirm this assumption^6^.

Our results match the existing, conflicting literature of 2D:4D and transgender identity where significant results have been observed partly in FtM, but not MtF individuals^42,43^, and partly in MtF, but not FtM individuals^44,45^. This emphasizes the necessity of meta-analytical reviews in this research area.

## Study 2: a meta-analysis

### Materials and methods

#### Search strategy

A two-step literature search was conducted using Google Scholar and PubMed and including abstracts in English from study inception until December 10, 2018. In the course of the review process we updated this literature search from study inception until February 28, 2020. The following search terms were combined in several ways: *2D:4D, 2nd to 4th digit ratio, second to fourth digit ratio, second-to-fourth finger length ratio, index and ring digit length ratio, transgender, transsexual, gender-variant identity, gender identity disorder, gender dysphoria, transsexualism, Harry Benjamin’s syndrome, gender divers,* and *gender nonconforming*. The reference lists of retrieved articles were searched manually in the second step. All abstracts were screened applying the selection criteria. The remaining articles were checked for eligibility according to the Preferred Reporting Items for Systemic Reviews and Meta-analyses (PRISMA) statement^46^ on the basis of a full-text review.

#### Study selection

We included case–control, cohort, and cross-sectional studies. The eligibility criteria were defined analogously to our meta-analytic review on 2D:4D and substance and computer use^13^ and are detailed in our coding protocol (Supplementary Table S5). The literature search was summarized according to the PRISMA guidelines^47^.

#### Data extraction

All recorded variables can be found in the previously defined coding protocol (Supplementary Table S5).

Data extraction and coding were performed by one investigator (E.-M.S.) and one research assistant (C.R.), independently of one another. Disagreement was resolved by discussion and agreeing upon the extracted values. Regarding quality assessment, the extractors’ values were averaged. Following our approach in Siegmann et al.^13^, we assessed the risk of bias with either the Newcastle–Ottawa Scale for case–control studies^48^ or an adaptation of the Newcastle–Ottawa Scale for cohort studies^48^, which was specifically designed for cross-sectional studies by Herzog et al.^49^ in their systematic review.

#### Statistical analysis

All analyses were conducted and all figures were made using the metafor package^50^ within the open-source software environment R, version 3.4.2^51^.

We estimated the standardized mean difference (Hedges’ g) in 2D:4D among MtF individuals and male controls in addition to among FtM individuals and female controls. In this analysis, a more feminized 2D:4D among MtF individuals compared to male controls is reflected by a positive Hedges’ g, whereas a more masculinized 2D:4D among FtM individuals compared to female controls is reflected by a negative Hedges’ g. Furthermore, we tested whether these mean differences were more pronounced among transgender individuals who had been reliably diagnosed by a clinician. For this purpose, we split the sample into transgender patients diagnosed by a clinician using common classification systems (DSM-5, DSM-IV(-TR), ICD-10) versus transgender patients not diagnosed by a clinician. The group of transgender individuals not diagnosed by a clinician consisted of (1) people subthreshold for the diagnosis, (2) people identifying as gender-variant, assessed with a shortened adaptation of the cross gender questionnaire^52^, (3) people identifying as tomboys, and (4) studies reporting correlative data. Correlative data were transformed into Hedges’ g using common transformation formulas^53^.

Moreover, we estimated Hedges’ g in the difference of R2D:4D and L2D:4D (2D:4Dr-l) among transgender individuals and controls since low 2D:4Dr-l values have been associated with high prenatal testosterone load^8^. We computed 2D:4Dr-l as the difference between the mean R2D:4D and mean L2D:4D, and related standard deviations were approximated by the pooled standard deviation of the R2D:4D and L2D:4D variances.

We performed univariate random-effects meta-analyses using restricted maximum likelihood estimations in which the point estimate for each study was weighted by the inverse of its variance. Non-independence among effect sizes was accounted for by aggregating, for example, in cases in which studies reported separate data for R2D:4D and L2D:4D. Two studies reported solely one 2D:4D value (right hand only^54^, dominant hand only^42^) entailing that our reported meta-analytic 2D:4D effect size refers to an aggregated 2D:4D rather than to M2D:4D. 2D:4Dr-l did not flow into this outcome measure. Heterogeneity among effect sizes within datasets was assessed using the I^2^ statistic.

We ran prespecified meta-regressions for the moderators study quality (i.e. the risk of bias in these studies), mean age of participants, and procedure of measuring 2D:4D (i.e., measurement by multiple independent raters, multiple times by one rater, once by one rater, or by the participants themselves). Thereby, the slope of the meta-regression line (β coefficient) indicated the strength of the association between the moderator and outcome. All meta-regressions were Bonferroni-corrected for multiple testing.

Moreover, we performed prespecified subgroup analyses to investigate the difference in the outcome measures between the right and the left hand, the different methods of measuring 2D:4D (i.e., with or without soft-tissue deformation) and the classification systems [i.e., DSM-5 vs. DSM-IV(-TR) and ICD-10 vs. DSM-IV(-TR)]. A comparison of DSM-5 versus ICD-10 was not possible due to small group sizes. “Measurement without soft-tissue deformation” comprised X-rays and direct measurement from the participants’ palm, and “measurement with soft-tissue deformation” comprised photocopies and hand scans. It is a prominent finding in 2D:4D research that associations with target traits are more pronounced in the right than in the left hand e.g.^55,56^. Recent meta-analyses on 2D:4D and gender identity^6,19^ report descriptive differences (insofar that the association is stronger with R2D:4D), but did not test for significant differences in these subgroups. We ran one post-hoc subgroup analysis examining differences between studies where the 2D:4D raters have been blinded vs. not blinded to the participants’ gender identity.

Small study effects were assessed by visual detection of asymmetries in contour-enhanced funnel plots and by the Egger regression test^57^. Following the authors’ original proposition, we considered analyses to be biased if the intercept differed from zero at *p* = 0.10^57^. We evaluated the sensitivity of our analysis by comparing models with and without effects that we assume to be influential outliers^58^. On the one hand, we defined statistically influential outliers following the approach by Viechtbauer and Cheung^58^: a study may be considered to be influential if at least one of the following is true: (1) the absolute DFFITS value is larger than 3√(p/(k − p)), where p is the number of model coefficients, and k the number of studies. (2) The lower tail area of a Chi-squared distribution with p degrees of freedom cut off by the Cook’s distance is larger than 50%. (3) The hat value is larger than 3(p/k). (4) Any DFBETAS value is larger than 1^58^. On the other hand, we considered studies to be influential outliers when the sample was substantially divergent to all other samples included. This is the case for one study with two child samples^59^. The literature is controversial as to whether symptoms of gender identity disorder in children persist into adulthood^45,60,61^, indicating that these samples potentially deviate from the rest of the studies included in our analysis.

*p* < 0.05 (2-sided) was considered statistically significant, except for the Egger test^57^ as stated above.

### Results

#### Eligible studies

The literature search is summarized in the PRISMA flow chart (Supplementary Figure S1). We identified 12 articles^6,42–45,54,59,62–66^ comprising 16 independent samples. Additionally, we conducted one investigation by ourselves described in the first part of this manuscript (Study 1: new original data). This results in 13 eligible articles added up to 17 independent samples. The characteristics of all included studies are detailed in the Supplementary Table S6 (online supplement).

#### Meta-analytic results

**Figure 1:**
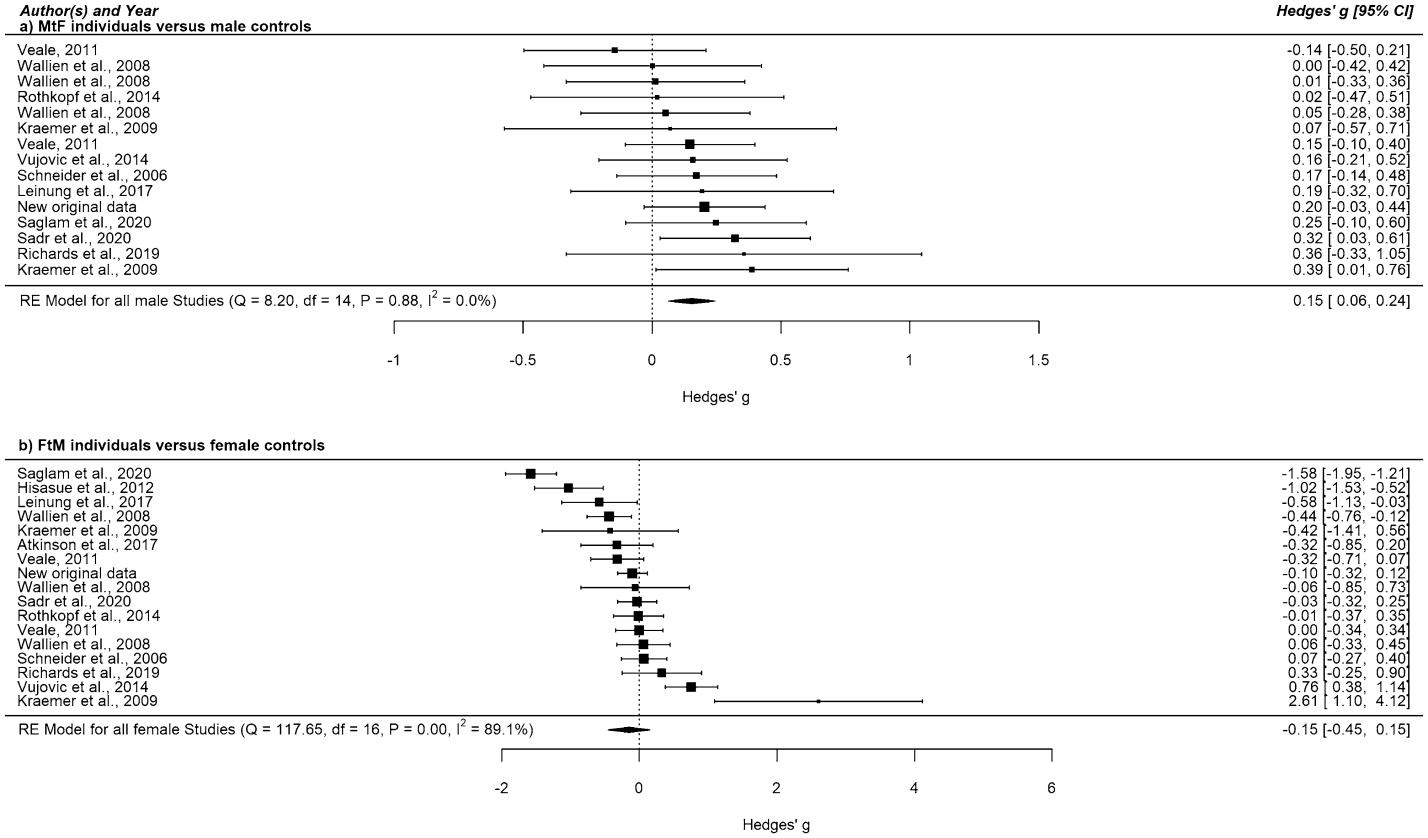
Forest plot of the standardized mean difference in 2D:4D among MtF individuals versus male controls and among FtM individuals versus female controls. This figure was created using R software^51^ (v3.4.2; https://www.R-project.org/). *CI* confidence interval, *FtM* female-to-male transgender, *MtF* male-to-female transgender, *RE Model* restricted maximum likelihood estimation model.

**Figure 2:**
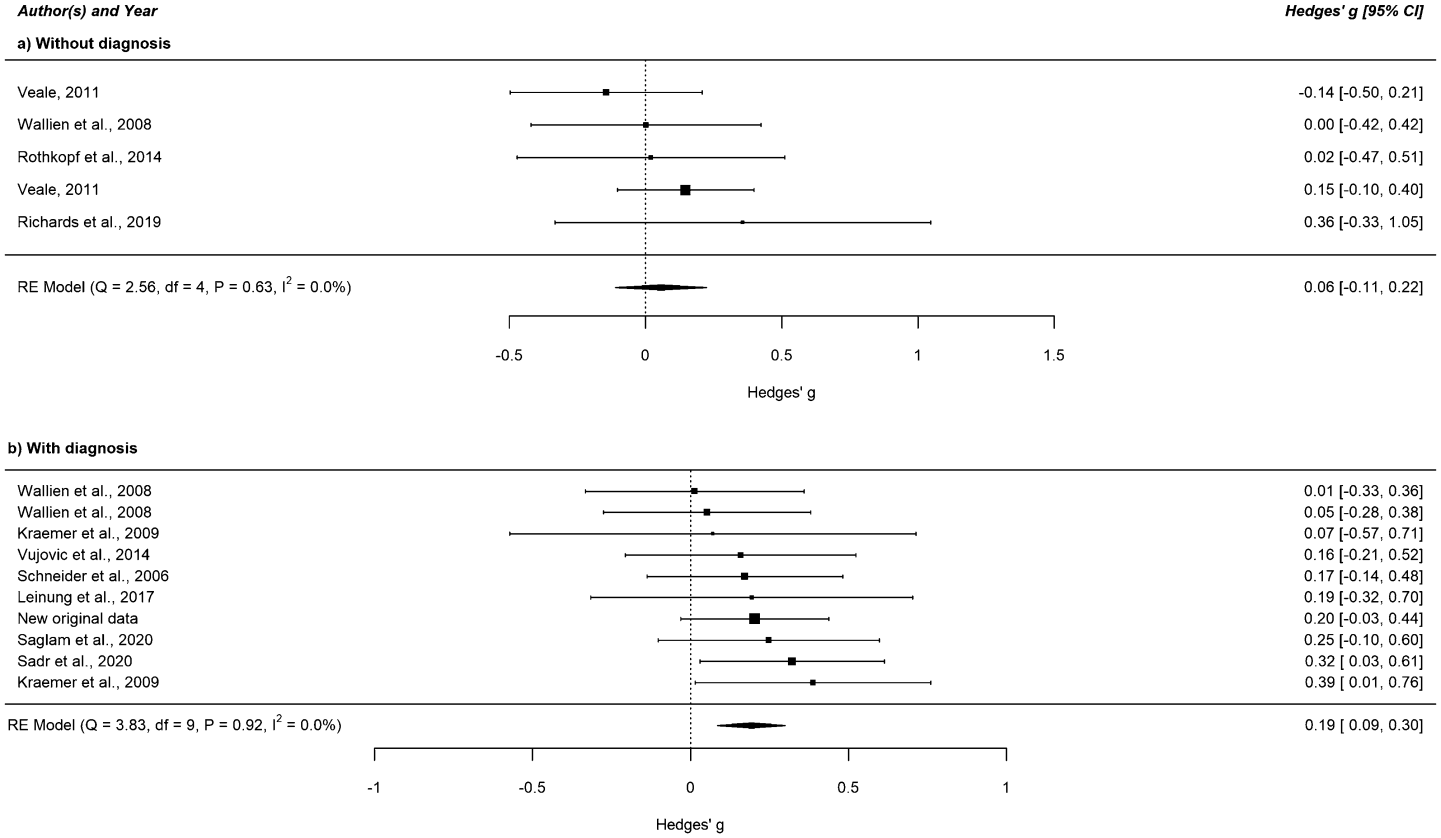
Forest plot of the standardized mean difference in 2D:4D among MtF individuals versus male controls separated according to whether diagnoses have been made (or not) by a clinician. This figure was created using R software^51^ (v3.4.2; https://www.R-project.org/). *CI* confidence interval, *MtF* male-to-female transgender, *RE Model* restricted maximum likelihood estimation model.

**Figure 3:**
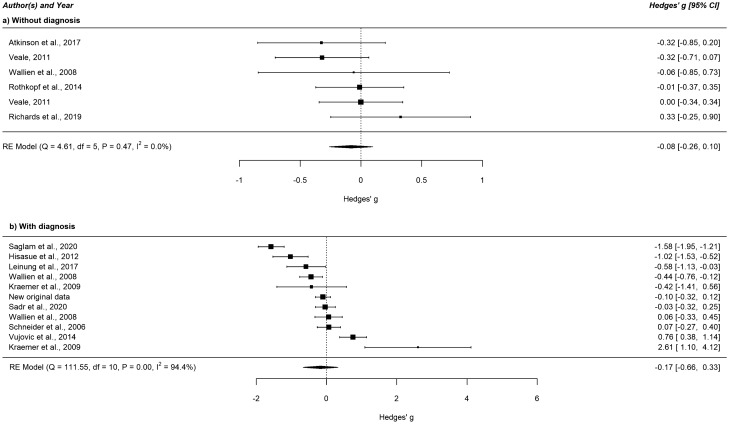
Forest plot of the standardized mean difference in 2D:4D among FtM individuals versus female controls separated according to whether diagnoses have been made (or not) by a clinician. This figure was created using R software^51^ (v3.4.2; https://www.R-project.org/). *CI* confidence interval, *FtM* female-to-male transgender, *RE Model* restricted maximum likelihood estimation model.

The meta-analytic results are shown in Table 2 and Figs. 1, 2 and 3. In line with our first hypothesis, we found that the aggregated 2D:4D was larger in MtF individuals than in male controls. This effect was even more pronounced in MtF individuals diagnosed by a clinician versus male controls, thus supporting our second hypothesis. We did not detect any significant meta-analytical differences between FtM individuals versus female controls, irrespective of a confirmed diagnosis. Concerning 2D:4Dr-l, none of the meta-analyses revealed any significant association with transgender identity (data not shown).

**Table 2:** Meta-analytic results: The standardized mean difference between transgender individuals and controls.

	MtF individuals versus male controls		FtM individuals versus female controls	
No. of subjects analyzed	1815		1859	
Hedges’ g (95% CI)	**0.153 (0.063; 0.243)**		− 0.152 (− 0.453; 0.149)	

	Diagnosis made by a clinician		Diagnosis made by a clinician	
	Yes	No	Yes	No
No. of subjects analyzed	1206	609	1298	561
Hedges’ g (95% CI)	**0.193 (0.086; 0.300)**	0.057 (− 0.111; 0.224)	− 0.166 (− 0.664; 0.333)	− 0.081 (− 0.261; 0.098)

*CI* confidence interval, *FtM* female-to-male transgender, *MtF* male-to-female transgender, *No* number.

#### Meta-regressions and subgroup analyses

None of the meta-regression analyses concerning study quality, mean age, and the procedure of measuring 2D:4D showed any significant effects with a Bonferroni-corrected threshold of *p* = 0.017. In subgroup analyses, effect sizes did not differ between 2D:4D measured on the right or the left hand, 2D:4D measured with or without soft tissue deformation, diagnoses made according to DSM-IV(-TR), ICD-10, or DSM-5, and between studies where 2D:4D raters have been blinded or not to the subjects’ case–control status. The exact figures are detailed in the Supplementary Table S7 (online supplement). As mentioned above (“Meta-analytic results” section), the subgroup analyses for our second hypothesis revealed significant differences.

#### Small study effects and sensitivity analyses

Concerning MtF individuals vs. male controls, the Egger test (z = −0.352, *p* = 0.725) and funnel plot (Supplementary Figure S2, online supplement) showed no evidence of small study effects. The funnel plot (Supplementary Figure S3, online supplement) and Egger test (z = 1.837, *p* = 0.066) regarding FtM individuals vs. female controls, however, revealed asymmetry in the published literature. Nevertheless, the applied trim and fill method^67^ suggested to compensate for this possible bias by adding 0 studies (SE = 2.571). Sensitivity analyses revealed no statistically influential outliers. When excluding one study examining two samples of children^59^, the effect size for FtM individuals versus female controls did not change substantially, whereas the effect size for MtF individuals versus male controls increased [Hedges’ g = 0.172; 95% CI (0.076; 0.268)].

### Discussion

In accordance with our hypotheses, we found associations of small effect size between feminized 2D:4D and MtF gender identity that were more pronounced in MtF individuals diagnosed by a specialized clinician. No significant results have been revealed in FtM individuals compared to female controls. Between-study heterogeneity was small for the MtF versus male sample (I^2^ = 0%) but high for the FtM versus female comparison (I^2^ = 89.1%), possibly resulting in slightly overestimated effect sizes in the latter sample. The high heterogeneity could not be explained by the pre-specified moderators since none of the meta-regression and subgroup analyses revealed any significant results. This contradicts the prominent 2D:4D findings concerning the influence of the measurement with or without soft tissue deformation on the relationship between 2D:4D and target traits^10,11^ and of more pronounced effects in the right compared to the left hand due to possible increased sensitivity of the right hand to prenatal testosterone exposure^44,68^. We also did not find a significant association between 2D:4Dr-l and transgender identity, which suggests that 2D:4Dr-l is not as suitable as 2D:4D to indicate relationships in this research area. Sensitivity analysis showed that the influence of single studies on the overall effect should be estimated minor.

## General discussion

Both the original study and the meta-analysis support our hypothesis that MtF individuals have a higher (i.e. feminized) 2D:4D than male controls. The effect was amplified when diagnoses were made according to classification criteria of gender dysphoria or transsexualism instead of self-identifying as transgender. This indicates that in males a lower prenatal androgen load associates with more pronounced gender dysphoria. In terms of practical relevance, the small effect sizes of up to Hedges’ g = 0.193 mean that the 2D:4D of 58% of male controls is below the average 2D:4D of MtF individuals^69^. Evaluation of the practical relevance by comparing our results to other meta-analyses in the research field of 2D:4D shows that effect sizes of approximately standardized mean difference = 0.2 are common, e.g. in addictive disorders^13^ or physical prowess^18^, but, rarely, higher effects exist as well (e.g. in sexual orientation of women^16^ and autism spectrum disorders^14^). However, with a non-overlap of 14.7% between transgender individuals and controls, one has to bear in mind that the practical impact is small, too^69^.

Nevertheless, our analysis implicates that prenatal androgen exposure plays a role in the development of male gender identity. A possible underlying mechanism may be a discrepancy between the two pre-/perinatal testosterone secretion peaks in males: (1) the first between 10 and 20 weeks of gestation entailing bodily changes and (2) the second during the perinatal window entailing organizational effects in the brain^43,70^, which can result in a discrepancy of sex between body and brain^71^. The lack of the second testosterone secretion peak in female infants with rather constant testosterone concentrations during the first year of life^70^ might explain why we did not detect any significant associations between 2D:4D and female gender identity. Considering anatomical features of the human brain, the BNST has been linked to gender identity^4^. An animal study examining mice^3^ revealed that perinatal metabolites of testosterone masculinize sexually dimorphic cell survival in the BNST, thus suggesting a possible association of perinatal testosterone and gender identity.

A previous meta-analysis^19^ reported significant associations of male transgender identity only with R2D:4D but not with L2D:4D, whereas a more recent meta-analysis^6^ found significant differences between MtF individuals and male controls in both R2D:4D and L2D:4D. Our original investigation, however, revealed significant associations with only L2D:4D but not with M2D:4D or R2D:4D. Our meta-analysis, in turn, points to an association of male transgender identity with (aggregated) 2D:4D in general with subgroup analyses revealing no significant difference between the meta-analytic effect sizes of R2D:4D and L2D:4D. Following these results, we cannot confirm an increased sensitivity to prenatal testosterone exposure in the right hand as often postulated^44,68^.

In order to draw conclusions about the prenatal androgen load, 2D:4D is used as a proxy^8^, as explained in the “Introduction” section. Recently, criticism has emerged on the use of 2D:4D as a biomarker of prenatal testosterone^72–75^. This is—among other things—due to a lack of X-linked inheritance in classical genetic studies^76,77^ or in genome-wide association studies (GWAS)^78^, even though the androgen receptor is located on the X chromosome. Some authors argue that if 2D:4D reflects prenatal testosterone effects and if androgen receptor variants moderate testosterone effects, androgen receptor variants should show systematic relationships with 2D:4D (for a more detailed explanation, see Hönekopp^79^). Additionally, meta-analyses do not confirm an association between 2D:4D and the effectiveness of androgen receptors (genetic CAG and GGC repeat length polymorphism)^72,79^. The largest GWAS on 2D:4D so far only shows a slight positive influence of CAG repeats in women, but not in men^78^. Thus, other mechanisms may also be involved in the development of 2D:4D besides sex hormones and genetics, for example prenatal corticosterone^80^ and prenatal stress^23,25,81^.

### Strengths and limitations

This analysis provides new case–control data from a comparably large sample size and a meta-analysis including a wide array of dimensions of transgender identity, which we incorporated in separate subgroup analyses. We ensure a standardized procedure for our meta-analysis by following the PRISMA statement entirely^46,47^ and by assessing the risk of bias of the studies according to the Newcastle–Ottawa Quality Assessment Scale for case–control and cohort studies^48^. For the sample of MtF individuals versus male controls, heterogeneity was small, indicating low inconsistency of studies’ results. This includes variation mainly due to sampling error, i.e. to the variation in study outcomes between studies. Our sensitivity analysis suggests that the effect does not depend on any one single study and therefore, is reasonably robust.

There are some limitations to this study. First, criticism of the concept of 2D:4D as an appropriate proxy for prenatal androgen exposure has emerged as described above in the “General discussion” section, and it is presumable that other mechanisms beside sex hormones and genetics also influence the development of 2D:4D. Second, despite its large sample size, the original investigation is still underpowered and thus, could not detect effects comparable to those described in a previous meta-analysis^19^. This additionally entails that our sample is not fully suited for multiple testing and we, consequently, could not control for alpha inflation; hence impeding the explanatory power of our results. Third, in the meta-analysis of FtM individuals versus female controls, heterogeneity was high, indicating systematic variation not caused by the study-related sampling errors and possibly resulting in overestimated effect sizes for this sample. Fourth, there were some methodological difficulties, such as information and selection bias potentially inflating the case–control difference and impeding the generalizability of results, when applying the study design of case–control studies. This extends to the control self-selection via flyers in the original investigation, an approach in which motivational factors related to personality traits or lifestyle are likely to play a role^82^. Consequently, the control group does not reflect a population-based sample.

### Future research

Following our meta-analysis, no recommendation can be made as to whether future 2D:4D examinations should be performed analyzing right or left hand 2D:4D or using methods with or without soft tissue deformation (for a definition, see Methods section). We recommend conducting original investigations in this field following a case–control design with reliable diagnoses (preferably made by clinicians) and computing a priori power analyses for determining an adequate sample size.

In summary, our results strengthen the assumption of a multifactorial etiology of gender identity and suggest that prenatal androgen levels are involved. Nevertheless, deeper analyses of other biological and non-biological mechanisms are required. We therefore recommend further studies on the developmental pathways of 2D:4D and transgender identity.

## Supplementary information

Supplementary Information.

## Data Availability

The datasets generated during and/or analyzed during the current study are not publicly available due to data protection limitations but are available from the corresponding author on reasonable request.
