# Supplementary material for: Digit ratio (2D:4D) and transgender identity: new original data and a meta-analysis
Source: Sci Rep. 2020 Nov 9;10:19326. doi: 10.1038/s41598-020-72486-6 (PMC7653965; doi:10.1038/s41598-020-72486-6)
Supplement: Supplementary file 1 — Supplementary Information. [file 41598_2020_72486_MOESM1_ESM.pdf]

Online supplement to the article

Digit ratio (2D:4D) and transgender identity: new original data and a meta-analysis

Eva-Maria Siegmann*[1], Tobias Müller[1,2], Isabelle Dziadeck[1], Christiane Mühle[1], Bernd Lenz[†,1,3], Johannes Kornhuber [†,1]

[1] Department of Psychiatry and Psychotherapy, Friedrich-Alexander University Erlangen-Nürnberg (FAU), Germany.

[2] Psychiatric Practice, Treibberg 5, 90403 Nuremberg, Germany

[3] Department of Addictive Behavior and Addiction Medicine, Central Institute of Mental Health (CIMH), Medical Faculty Mannheim, Heidelberg University, Germany.

[†]Bernd Lenz and Johannes Kornhuber should be considered joint senior author.

*Corresponding author at: Department of Psychiatry and Psychotherapy, Friedrich-Alexander University Erlangen-Nürnberg (FAU), Schwabachanlage 6, D-91054 Erlangen, Germany, Phone: +49 9131 85-34166, E-Mail: eva-maria.siegmann@uk-erlangen.de

Table of contents

Supplementary Table S1. Cronbach's alpha values for GIDYQ and UGDS 3

Supplementary Table S2. Pearson correlations of gender dysphoria scale scores with 2D:4D 4

Supplementary Table S3. Pearson correlations between 2D:4D and sexual orientation in transgender individuals 5

Supplementary Table S4. Frequencies of early and late onset gender dysphoria 5

Supplementary Table S5. Coding protocol 6

Supplementary Table S6. Characteristics of all included studies. 13

Supplementary Table S7. Results of the meta-regression and subgroup analyses 16

Supplementary Figure S1. PRISMA flow chart 17

Supplementary Figure S2. Funnel plot of the meta-analysis comparing 2D:4D among MtF individuals versus male controls 18

Supplementary Figure S3. Funnel plot of the meta-analysis comparing 2D:4D among FtM individuals versus female controls 19

Supplementary Table S1. Cronbach's alpha values for GIDYQ and UGDS

	**GIDYQ**	**UGDS**
**MtF individuals** (n = 96)	α = 0.647	α = 0.815
**Male controls** (n = 101)	α = 0.294	α = 0.283
**FtM individuals** (n = 136)	α = 0.348	α = 0.516
**Female controls** (n = 102)	α = 0.671	α = 0.663

Abbreviations: FtM, female-to-male transgender; GIDYQ, Gender Identity/Gender Dysphoria Questionnaire; MtF, male-to-female transgender; UGDS, Utrecht Gender Dysphoria Scale.

Supplementary Table S2. Pearson correlations of gender dysphoria scale scores with 2D:4D

	Male-to-female					Male					Female-to-male					Females				
		GIDYQ[a]		UGDS			GIDYQ[a]		UGDS			GIDYQ[a]		UGDS			GIDYQ[a]		UGDS	
	N	*r*	*P*	*r*	*P*	*N*	*r*	*P*	*r*	*P*	*N*	*r*	*P*	*r*	*P*	*N*	*r*	*P*	*r*	*P*
M2D:4D	95	-0.204	**.048**	-0.134	.197	101	0.135	.179	-0.166	.098	135	0.007	.937	-0.026	.767	102	0.027	.790	-0.027	.791
R2D:4D	96	-0.206	**.044**	-0.147	.152	101	0.044	.665	-0.104	.301	136	0.016	.851	-0.018	.836	102	0.057	.566	-0.038	.707
L2D:4D	95	-0.177	.086	-0.107	.302	101	0.202	**.043**	-0.202	**.043**	135	-0.008	.924	-0.029	.740	102	-0.006	.952	-0.012	.902
2D:4Dr-l	95	-0.039	.705	-0.052	.618	101	-0.229	**.021**	0.148	.139	135	0.040	.642	0.015	.862	102	0.080	.421	-0.031	.758

P < 0.05 in bold, [a]reversed. GIDYQ, Gender Identity/Gender Dysphoria Questionnaire; UGDS, Utrecht Gender Dysphoria Scale; 2D:4D, second-to-fourth finger length ratio; M2D:4D, mean of R2D:4D and L2D:4D; R2D:4D, right-hand 2D:4D; R2D, length of the right-hand index finger; R4D, length of the right-hand ring finger; L2D:4D, left-hand 2D:4D; L2D, length of the left-hand index finger; L4D, length of the left-hand ring finger; 2D:4Dr-l, difference between R2D:4D and L2D:4D.

Supplementary Table S3. Pearson correlations between 2D:4D and sexual orientation in transgender individuals

		L2D:4D	R2D:4D	M2D:4D	2D:4Dr-l
**MtF individuals**	r	-0.040	-0.069	-0.059	-0.043
	p	0.708	0.514	0.582	0.691
	n	90	91	90	90
**FtM individuals**	r	0.116	0.036	0.084	-0.111
	p	0.185	0.679	0.338	0.203
	n	133	134	133	133

Abbreviations: 2D:4Dr-l, difference between right and left hand 2D:4D; FtM, female-to-male transgender; L2D:4D, left hand 2D:4D; M2D:4D, mean of right and left hand 2D:4D; MtF, male-to-female transgender; n, sample size; R2D:4D, right hand 2D:4D.

Supplementary Table S4. Frequencies of early and late onset gender dysphoria

	MtF individuals		FtM individuals	
	**Early**	**Late**	**Early**	**Late**
**Question 1**	40.0%	17.3%	66.2%	5.9%
**Question 2**	57.3%	12.7%	75.5%	4.6%

Annotations: Questions could be answered with “yes”, “no”, or “I don’t know”. Question 1: “Did you identify with the other gender before puberty?”. Question 2: “Have you already experienced discomfort with your own gender during childhood?”

Abbreviations: FtM, female-to-male transgender; MtF, male-to-female transgender.

Supplementary Table S5. Coding protocol

Coding protocol

2D:4D and transgender identity

Inclusion criteria:

a) Year of publication: 1983-2020
b) Publication language: English
c) Digit ratio was measured as continuous measure
d) Cases (transgender persons) and controls were compared concerning their digit ratio OR the correlation of digit ratio and any form of transsexuality / transgender identity (measured continuously) was examined OR both
e) In case-control studies, controls were not diagnosed with any form of transsexuality or gender dysphoria
f) Effect sizes or associated data to compute effect sizes are reported

Exclusion criteria:

a) Abstracts or pilot data
b) Publication language other than English
c) Digit ratio was measured as a categorical measure
d) Only partial correlations or β-coefficients from multiple regression models are reported

Coding procedure:

(a) One line represents one effect size

(b) If effect sizes are reported separately for the whole sample and for subgroups, only information concerning the whole sample will be extracted. If information is only reported for subgroups, every subgroup will be treated as distinct sample (variable sno).

(c) If there are multiple effect sizes within one sample concerning different outcome variables (e.g. different ways of measuring 2D:4D), every effect size will be reported in its own line. These effect sizes are coded as dependent by allocating the same number for the variable sno.

(d) No computations should be carried out while coding. Information is extracted directly without conversions.

Variable	Description	Code	Example
General and sample characteristics			
`study`	Study name comprising lead author and year of publication.	Free specification	Manning2000<br>Manning2000a
`Pubyear`	Year of publication	Range: [1983, 2018]	2007
`Incl`	Effect size can or cannot be included in statistical analysis	0 = exclude<br>1 = include	1
`Sid`	Consecutive number for every publication	Range: [1, ∞]	1
`Sno`	Consecutive number for every sample	Range: [1, ∞]	1
`Colyear`	Year of conduct	Range: [1983, 2018]	2007
`Cntry`	Country of conduct<br>If not reported, extract affiliation of lead author as ISO-CODE 2:<br>http://en.wikipedia.org/wiki/ISO_3166-1_alpha-2<br>or „XX" describing samples with participants originating from different countries	Free specification	DE
`Pubtype`	Publication type	1 = Peer-reviewed Journal<br>2 = Book<br>3 = Thesis (Master / PhD)<br>4 = Poster<br>5 = Other	1
`N`	Sample size *N*	Range: [2, ∞]	100
`Samtype`	Description of sample (coded)	1 = children<br>2 = students (undergraduates, college)<br>3 = Adults, mixed sample	2
`Age`	Mean age (in years)	Range: [0, ∞]	16.86

Variable	Description	Code	Example
	Effect sizes (1=male; 2=male-to-female; 3=female; 4=female-to-male)		
n1	Sample size of male sample	Range: [2, ∞]	100
n2	Sample size of male-to-female sample	Range: [2, ∞]	100
n3	Sample size of female sample	Range: [2, ∞]	100
n4	Sample size of female-to-male sample	Range: [2, ∞]	100
m1	Male mean 2D:4D	Range: [0, ∞]	0.9
sd1	Male standard deviation of 2D:4D	Range: [0, ∞]	0.1
cilow1	Lower limit of the 99% confidence interval reported for mean 2D:4D of the male sample	Range: [0, ∞]	0.8
ciup1	Upper limit of the 99% confidence interval reported for mean 2D:4D of the male sample	Range: [0, ∞]	1.0
m2	Male-to-female mean 2D:4D	Range: [0, ∞]	0.9
sd2	Male-to-female standard deviation of 2D:4D	Range: [0, ∞]	0.1
cilow2	Lower limit of the 99% confidence interval reported for mean 2D:4D of the male-to-female sample	Range: [0, ∞]	0.8
ciup2	Upper limit of the 99% confidence interval reported for mean 2D:4D of the male-to-female sample	Range: [0, ∞]	1.0
m3	Female mean 2D:4D	Range: [0, ∞]	0.9
sd3	Female standard deviation of 2D:4D	Range: [0, ∞]	0.1
cilow3	Lower limit of the 99% confidence interval reported for mean 2D:4D of the female sample	Range: [0, ∞]	0.8
ciup3	Upper limit of the 99% confidence interval reported for mean 2D:4D of the female sample	Range: [0, ∞]	1.0
m4	Female-to-male mean 2D:4D	Range: [0, ∞]	0.9

Variable	Description	Code	Example
Effect sizes (1=male; 2=male-to-female; 3=female; 4=female-to-male)			
sd4	Female-to-male standard deviation of 2D:4D	Range: [0, ∞]	0.1
cilow4	Lower limit of the 99% confidence interval reported for mean 2D:4D of the female-to-male sample	Range: [0, ∞]	0.8
ciup4	Upper limit of the 99% confidence interval reported for mean 2D:4D of the female-to-male sample	Range: [0, ∞]	1.0
n_m	**Only in the case of correlative data**: Sample size of male sample	Range: [2, ∞]	100
n_f	**Only in the case of correlative data**: Sample size of female sample	Range: [2, ∞]	100
r_m	**Only in the case of correlative data**: Pearson's correlation coefficient r for the male sample	Range: [-1, 1]	0.5
r_f	**Only in the case of correlative data**: Pearson's correlation coefficient r for the female sample	Range: [-1, 1]	0.5

Variable	Description	Code	Example
	Additional information		
qual1	Category Selection<br>- Is the case definition adequate?<br>- Representativeness of cases<br>- Selection of controls<br>- Definition of controls	0 = 0 Stars<br>1 = 1 Star<br>2 = 2 Stars<br>3 = 3 Stars<br>4 = 4 Stars	1
qual2	Category Comparability: Comparability of cases and controls on the basis of the design or analysis<br>- Study controls for transsexuality<br>- Study controls for any additional factor	0 = 0 Stars<br>1 = 1 Star<br>2 = 2 Stars	1
qual3	Category Exposure<br>- Ascertainment of exposure<br>- Same method of ascertainment for cases and controls<br>- Non-response rate	0 = 0 Stars<br>1 = 1 Star<br>2 = 2 Stars<br>3 = 3 Stars	1
qual_r1	**Only in the case of correlative data:** Category Selection<br>- Representativeness of the sample<br>- Sample size<br>- Ascertainment of exposure<br>- Non-respondents	0 = 0 Stars<br>1 = 1 Star<br>2 = 2 Stars<br>3 = 3 Stars<br>4 = 4 Stars	1
qual_r2	**Only in the case of correlative data:** Category Comparability: Confounding factors are controlled<br>- The study controls for the most important factor (diseases influencing 2D:4D. e.g. fractures, hormonal abnormalities)<br>- The study controls for any additional factor	0 = 0 Stars<br>1 = 1 Star<br>2 = 2 Stars	1
qual_r3	**Only in the case of correlative data:** Category Outcome:<br>- Assessment of Outcome<br>- The statistical test used to analyze the data is clearly described and appropriate	0 = 0 Stars<br>1 = 1 Star<br>2 = 2 Stars	1

Variable	Description	Code	Example
	Additional information		
`method`	Method of measuring 2D:4D	1 = x-ray<br>2 = photocopy or scans<br>3 = directly from subjects' hand	1
`rating`	Quality of 2D:4D measurement: Have there been taken actions to assure the reproducibility of the 2D:4D value that was used for computation?	1 = multiple independent raters<br>2 = one rater performing multiple measurements<br>3 = one rater performing one measurement<br>4 = self-measurement by participants	1
`hand`	Subjects' hand used to compute 2D:4D	1 = right hand only<br>2 = left hand only<br>3 = mix of both hands	1
`diagnosis`	Has the diagnosis of gender dysphoria or gender identity disorder been made or verified by a clinician?	0 = no<br>1 = yes	1
`system`	**Only in the case of diagnosis = 1**: Which classification system has been applied when diagnosing gender dysphoria or gender identity disorder?	1 = ICD-10<br>2 = DSM-IV(-TR)<br>3 = DSM-5<br>4 = other	1
`masking`	Have all raters been blinded regarding case-control status when they recorded the digit ratio?	0 = no<br>1 = yes	1

Supplementary Table S6. Characteristics of all included studies.

	MtF individuals vs. male controls				FtM individuals vs. female controls			
First author, year of publication	N_{MtF}	2D:4D$_{MtF}$ (M ± SD)	N_{males}	2D:4D$_{males}$ (M ± SD)	N_{FtM}	2D:4D$_{FtM}$ (M ± SD)	$N_{females}$	2D:4D$_{females}$ (M ± SD)
Schneider et al., 2006[a,e,f,h]	63	0.967 ± 0.034	58	0.957 ± 0.029	43	0.975 ± 0.029	65	0.972 ± 0.028
Schneider et al., 2006[b,e,f,h]	63	0.953 ± 0.036	58	0.952 ± 0.026	43	0.964 ±0.032	65	0.963 ± 0.03
Wallien et al., 2008[a,e,f]	39	0.952 ± 0.029	89	0.955 ± 0.041	38	0.969 ± 0.05	112	0.978 ± 0.034
Wallien et al., 2008[b,e,f]	38	0.967 ± 0.035	89	0.96 ± 0.039	39	0.961 ± 0.034	112	0.982 ± 0.032
Wallien et al., 2008[a,e,f]	38	0.955 ± 0.04	71	0.953 ± 0.034	29	0.969 ± 0.051	70	0.967 ± 0.037
Wallien et al., 2008[b,e,f]	36	0.965 ± 0.037	67	0.966 ± 0.034	24	0.987 ± 0.061	70	0.983 ± 0.047
Wallien et al., 2008[a,e,g]	23	0.965 ± 0.052	71	0.953 ± 0.034	5	0.977 ± 0.028	70	0.967 ± 0.037
Wallien et al., 2008[b,e,g]	20	0.954 ± 0.055	67	0.966 ± 0.034	5	0.965 ± 0.013	70	0.983 ± 0.047
Kraemer et al., 2009[a,e,f]	15	0.961 ± 0.028	13	0.959 ± 0.041	14	0.982 ± 0.033	2	0.912 ± 0.025
Kraemer et al., 2009[b,e,f]	15	0.954 ± 0.032	13	0.951 ± 0.036	14	0.976 ± 0.023	2	0.9 ± 0.017
Kraemer et al., 2009[a,e,f]	24	0.97 ± 0.027	163	0.954 ± 0.032	3	0.96 ± 0.047	188	0.974 ± 0.033
Kraemer et al., 2009[b,e,f]	24	0.963 ± 0.036	163	0.954 ± 0.033	3	0.955 ± 0.016	188	0.97 ± 0.035
Veale, 2011[a,d,g]	117	0.989 ± NA	42	0.997 ± NA	38	0.981 ± NA	84	0.999 ± NA

First author, year of publication	N_{MtF}	2D:4D$_{MtF}$ (M ± SD)	N_{males}	2D:4D$_{males}$ (M ± SD)	N_{FtM}	2D:4D$_{FtM}$ (M ± SD)	$N_{females}$	2D:4D$_{females}$ (M ± SD)
Veale, 2011[a,d,g]	191	0.992 ± NA	90	0.984 ± NA	41	0.992 ± NA	154	0.992 ± NA
Hisasue et al., 2012[a,e,f]	NA	NA	NA	NA	37	0.955 ± 0.029	20	0.999 ± 0.035
Hisasue et al., 2012[b,e,f]	NA	NA	NA	NA	37	0.954 ± 0.036	20	0.979 ± 0.04
Vujovic et al., 2014[a,d,f,h]	42	0.916 ± 0.058	45	0.896 ± 0.075	38	0.93 ± 0.046	48	0.896 ± 0.05
Vujovic et al., 2014[b,d,f,h]	42	0.928 ± 0.041	45	0.927 ± 0.041	38	1.013 ± 0.189	48	0.903 ± 0.061
Atkinson et al., 2017[a,e,g]	NA	NA	NA	NA	19	0.967 ± 0.038	25	0.994 ± 0.038
Atkinson et al., 2017[b,e,g]	NA	NA	NA	NA	19	0.986 ± 0.043	25	0.984 ± 0.038
Leinung et al., 2017[c,d,f]	68	0.978 ± 0.029	19	0.972 ± 0.036	50	0.983 ± 0.027	18	0.998 ± 0.021
New original data, 2019[a,e,f]	110	0.963 ± 0.03	101	0.959 ± 0.028	151	0.972 ± 0.028	102	0.975 ± 0.03
New original data, 2019[b,e,f]	109	0.969 ± 0.029	101	0.961 ± 0.03	150	0.971 ± 0.029	102	0.974 ± 0.032
Richards et al., 2019[a,d,g]	13	0.997 ± 0.047	12	0.956 ± 0.046	20	1.002 ± 0.053	16	0.971 ± 0.046
Richards et al., 2019[b,d,g]	14	0.995 ± 0.048	12	1.003 ± 0.064	20	1.003 ± 0.058	16	1.000 ± 0.061
Saglam et al., 2020[a,e,f]	45	0.968 ± 0.013	58	0.973 ± 0.008	54	0.980 ± 0.010	58	0.998 ± 0.008
Saglam et al., 2020[b,e,f]	45	0.980 ± 0.010	58	0.970 ± 0.010	54	0.983 ± 0.008	58	0.990 ± 0.005
Sadr et al., 2020[a,e,f]	89	0.972 ± 0.029	56	0.959 ± 0.033	104	0.981 ± 0.030	53	0.983 ± 0.033
Sadr et al., 2020[b,e,f]	88	0.981 ± 0.033	56	0.974 ± 0.029	104	0.991 ± 0.034	53	0.991 ± 0.032

Correlative data				
	Male sample		Female sample	
	N_{males}	r_{males}	$N_{females}$	$r_{females}$
Rothkopf et al., 2014[a,d,g]	49	0.05	89	0.03
Rothkopf et al., 2014[b,d,g]	49	-0.03	89	-0.04

[a]Right 2D:4D
[b]Left 2D:4D
[c]2D:4D of the dominant hand
[d]No tissue deformation when measuring 2D:4D
[e]Tissue deformation when measuring 2D:4D
[f]Diagnosis made by a clinician
[g]Diagnosis not made by a clinician
[h]Data obtained from graphs

Supplementary Table S7. Results of the meta-regression and subgroup analyses

	MtF individuals vs. male controls	FtM individuals vs. female controls
**Meta-regression analyses**		
Study quality	*β* = 0.002 *P* = 0.973	*β* = 0.192, *P* = 0.265
Mean age	*β* = 0.002, *P* = 0.654	*β* = - 0.010, *P* = 0.627
Procedure of measuring 2D:4D	*β* = - 0.068, *P* = 0.072	*β* = - 0.036, *P* = 0.799
**Subgroup analyses**		
Right vs. left hand	*z* = 0.129, *P* = 0.897	*z* = 0.221, *P* = 0.825
With vs. without soft tissue deformation	*z* = - 0.899, *P* = 0.369	*z* = 0.954, *P* = 0.340
DSM-IV(-TR) vs. ICD-10	*z* = - 0.431, *P* = 0.666	*z* = 0.409, *P* = 0.682
DSM-IV(-TR) vs. DSM-5	*z* = 0.943, *P* = 0.346	*z* = - 1.044, *P* = 0.296
With or without blinding of 2D:4D raters	*z* = 0.353, *P* = 0.72	*z* = - 0.676, *P* = 0.499

Supplementary Figure S1. PRISMA flow chart

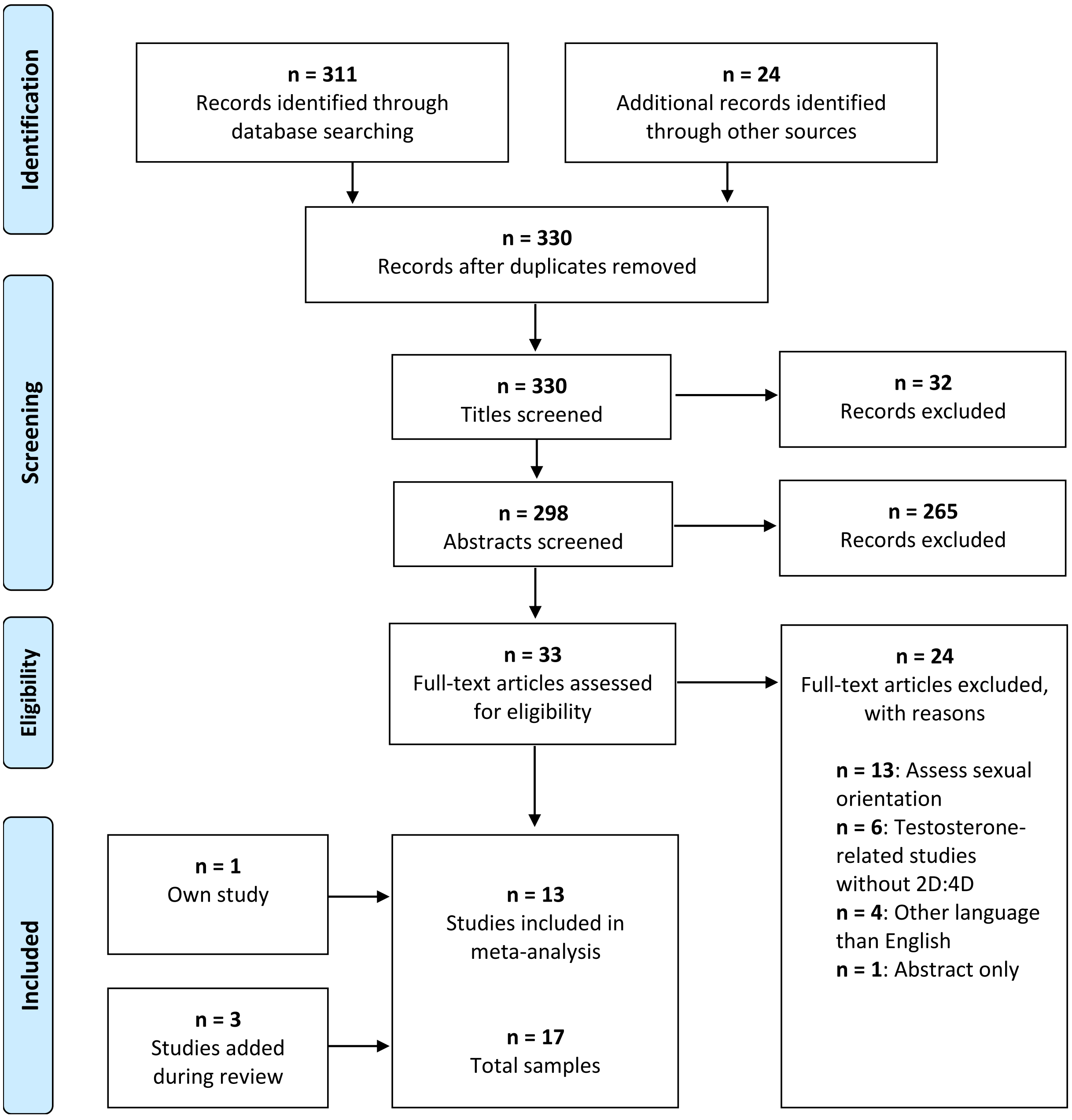

Supplementary Figure S2. Funnel plot of the meta-analysis comparing 2D:4D among MtF individuals versus male controls

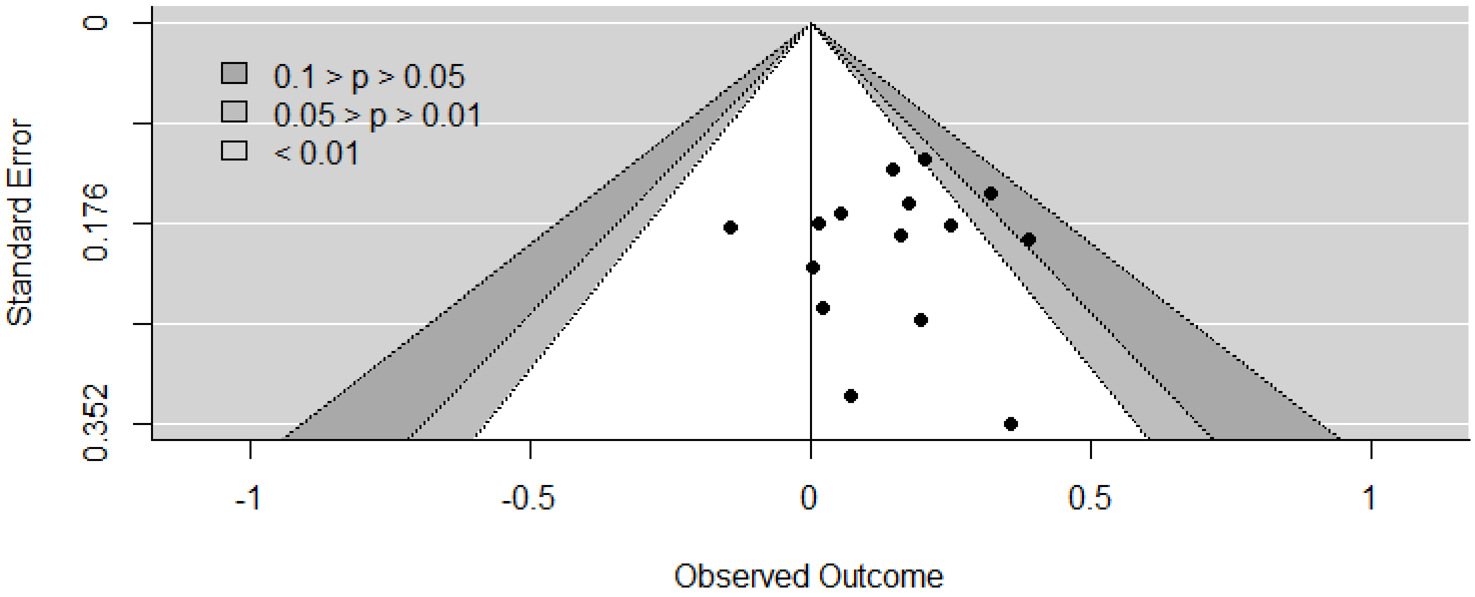

Annotation: This figure was created using R software (v3.4.2; https://www.R-project.org/).

Supplementary Figure S3. Funnel plot of the meta-analysis comparing 2D:4D among FtM individuals versus female controls

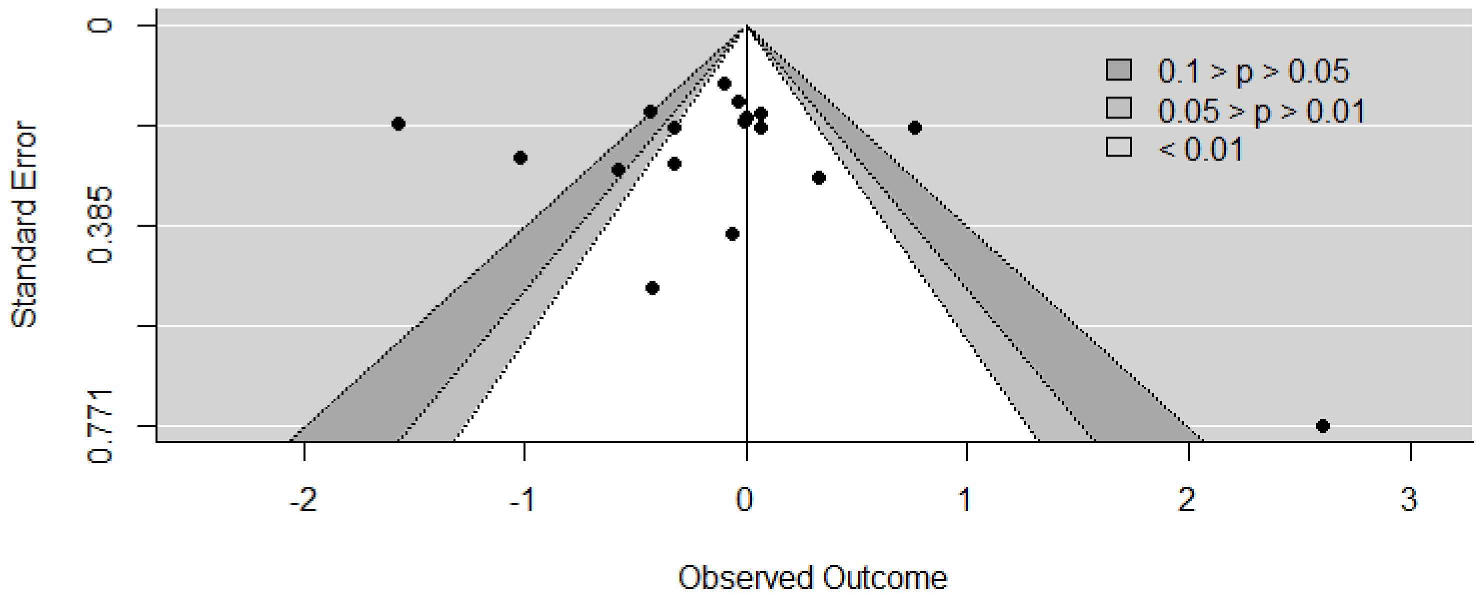

Annotation: This figure was created using R software (v3.4.2; https://www.R-project.org/).
